# Effect of Treatment Modality on the Hypothalamic–Pituitary Function of Patients Treated with Radiation Therapy for Pituitary Adenomas: Hypothalamic Dose and Endocrine Outcomes

**DOI:** 10.3389/fonc.2014.00073

**Published:** 2014-04-09

**Authors:** Andrew Elson, Joseph Bovi, Kawaljeet Kaur, Diana Maas, Grant Sinson, Chris Schultz

**Affiliations:** ^1^Department of Radiation Oncology, Medical College of Wisconsin, Milwaukee, WI, USA; ^2^Division of Endocrinology, Metabolism, and Clinical Nutrition, Medical College of Wisconsin, Milwaukee, WI, USA; ^3^Department of Neurosurgery, Medical College of Wisconsin, Milwaukee, WI, USA

**Keywords:** pituitary adenoma, tomotherapy, gamma knife, hypothalamus, endocrinopathy, IMRT, 3D conformal radiotherapy

## Abstract

**Background:** Both fractionated external beam radiotherapy and single fraction radiosurgery for pituitary adenomas are associated with the risk of hypothalamic–pituitary (HP) axis dysfunction.

**Objective:** To analyze the effect of treatment modality (Linac, TomoTherapy, or gamma knife) on hypothalamic dose and correlate these with HP-axis deficits after radiotherapy.

**Methods:** Radiation plans of patients treated post-operatively for pituitary adenomas using Linac-based 3D-conformal radiotherapy (CRT) (*n* = 11), TomoTherapy-based intensity modulated radiation therapy (IMRT) (*n* = 10), or gamma knife stereotactic radiosurgery (*n* = 12) were retrospectively reviewed. Dose to the hypothalamus was analyzed and post-radiotherapy hormone function including growth hormone, thyroid stimulating hormone, adrenocorticotropic hormone, prolactin, and gonadotropins (follicle stimulating hormone/luteinizing hormone) were assessed.

**Results:** Post-radiation, 13 of 27 (48%) patients eligible for analysis developed at least one new hormone deficit, of which 8 of 11 (72%) occurred in the Linac group, 4 of 8 (50%) occurred in the TomoTherapy group, and 1 of 8 (12.5%) occurred in the gamma knife group. Compared with fractionated techniques, gamma knife showed improved hypothalamic sparing for DMax Hypo and V12Gy. For fractionated modalities, TomoTherapy showed improved dosimetric characteristics over Linac-based treatment with hypothalamic DMean (44.8 vs. 26.8 Gy *p* = 0.02), DMax (49.8 vs. 39.1 Gy *p* = 0.04), and V12Gy (100 vs. 76% *p* = 0.004).

**Conclusion:** Maximal dosimetric avoidance of the hypothalamus was achieved using gamma knife-based radiosurgery followed by TomoTherapy-based IMRT, and Linac-based 3D conformal radiation therapy, respectively.

## Introduction

Radiotherapy plays an integral role in the management of patients with pituitary adenomas, and this includes both fractionated external beam radiotherapy (EBRT) and stereotactic radiosurgery (SRS). Fractionated EBRT and SRS both afford excellent local control rates for pituitary adenomas, generally on the order of 90% at 10 years (1–3). Both fractionated EBRT and SRS put patients at risk for functional endocrine deficits, which is both a dose and time dependent phenomenon (4). Such abnormalities include deficits in growth hormone (GH), prolactin (PRL), adrenocorticotropic hormone (ACTH), thyroid stimulating hormone (TSH), and gonadotropins follicle stimulating hormone (FSH) and luteinizing hormone (LH), with GH generally being the most radiosensitive endocrine axis (5). In comparison to fractionated EBRT, SRS affords a more convenient option for patients and is becoming a more frequently used modality of treatment (6). However, certain tumor and patient selection criteria may require the use of fractionated radiotherapy. For instance, at the Medical College of Wisconsin (MCW), the use of SRS is generally reserved for patients with pituitary tumors <3 cm in size and with at least 3 mm clearance from the optic chiasm (OC).

The hypothalamus plays a critical role in the functioning of the hypothalamic–pituitary (HP) axis, and radiation-induced injury is believed to affect both the pituitary and the hypothalamus (7). Doses to the hypothalamus as low as 12 Gy may be implicated in increasing the risk of radiotherapy associated endocrinopathies, and doses typically used in the treatment of brain tumors (>50 Gy) commonly result in HP-axis deficiency (8, 9). To better understand the relationship between hypothalamic dose and endocrine dysfunction, we reviewed patients undergoing treatment for pituitary adenomas at our institution. Hypothalamic dose characteristics were analyzed by treatment modality [Linac-based 3D-conformal radiotherapy (CRT), TomoTherapy-based intensity modulated radiation therapy (IMRT), and gamma knife SRS], and endocrine outcomes were assessed accordingly.

## Materials and Methods

### Patients

Between December 2005 and January 2012, 33 patients were identified with pituitary adenomas who underwent gamma knife SRS (*n* = 12), fractionated TomoTherapy-based IMRT (*n* = 10), or fractionated Linac-based 3D-CRT (*n* = 11). All aspects of this retrospective study were approved by the MCW Institutional Review Board. All patients analyzed had routine endocrine follow-up at MCW, and clinical data were obtained from the institutional electronic medical record. Dosimetric information was acquired through restoration of the original treatment plans maintained in the departmental database. Baseline patient characteristics are described in Table 1. Pre-radiotherapy endocrine deficits were present in 84.8% of patients, and 45% of patients had functioning adenomas vs. 55% non-functioning. Of all adenomas, 30% were GH secreting, 12% PRL secreting, and 3% ACTH secreting. Four patients were treated with medical therapy to oppose hypersecretory function prior to surgery (two patients treated with a dopamine agonist for PRL secretion in the TomoTherapy group, and two patients treated with octreotide for GH secretion in the gamma knife group). All patients had previously undergone endonasal transsphenoidal surgery. All patients were diagnosed with macroadenomas and underwent a subtotal resection except one patient in the Linac group who underwent a biopsy only. The median study follow-up period was 24 months (range 2–76 months).

**Table 1:** **Baseline patient characteristics**.

	Linac	Tomo Therapy	Gamma knife	Total
*N*	11	10	12	33
Male (%)	6 (54.5)	6 (60)	5 (41.5)	17 (51.5)
Female (%)	5 (45.5)	4 (40)	7 (58.3)	16 (48.5)
Mean age	43.8	49.2	46	44.4
Transsphenoidal resection (%)	100	100	100	100
Deficit prior to RT (%)	6 (54.5)	10 (100)	12 (100)	28 (84.8)
Non-functioning adenoma (%)	5 (45)	6 (60)	7 (58)	18 (55)
Functioning adenoma (%)	6 (55)	4 (40)	5 (42)	15 (45)
GH secreting (%)	5 (45)	1 (10)	4 (33)	10 (30)
PRL secreting (%)	0 (0)	3 (30)	1 (8)	4 (12)
TSH secreting (%)	0 (0)	0 (0)	0 (0)	0 (0)
ACTH secreting (%)	1 (9)	0 (0)	0 (0)	1 (3)

### Radiotherapy

Linac-based 3D-CRT was performed on one of three institutional linear accelerators. Plans were based on a five field non-coplanar design including an anterior “vertex” field requiring a 90° couch rotation. Radiation dose to the target volume ranged from 48.6 to 54 Gy. All 3D-CRT planning was performed using Xio radiation treatment planning software (Elekta/CMS, St Louis, MO, USA).

TomoTherapy-based IMRT was performed on the institutional TomoTherapy unit. IMRT planning was performed using the TomoTherapy radiation planning system (TomoTherapy, Madison, WI, USA). Radiation dose to the target volume ranged from 50 to 54 Gy.

Gamma knife SRS was performed using the Leksell Gamma knife (Elekta AB, Stockholm, Sweden). MRI imaging with a stereotactic head frame was acquired for treatment planning for each case, and planning was performed using Leksell GammaPlan software (Elekta AB, Stockholm, Sweden). Radiation dose to the target volume ranged from 12 to 20 Gy in a single fraction prescribed to the 50% isodose line (IDL).

In this study group at the time of treatment planning and delivery, the hypothalamus was not explicitly contoured, monitored, or identified as an avoidance structure for any of the three treatment techniques.

The determination of eligibility for SRS vs. fractionated EBRT was made primarily based on size of the tumor and distance from the OC, with a requirement of <3 cm tumor with >3 mm clearance from the OC preferred for SRS. The use of 3D-CRT vs. TomoTherapy IMRT was practitioner dependent and partially determined by the year of treatment and the availability of the modality, with a trend toward increased use of TomoTherapy during the later years of the study period. Linac-based IMRT was not performed due to the perceived improved conformality of TomoTherapy over Linac-based IMRT during the study period.

### Dosimetric analysis

In order to analyze hypothalamic dose characteristics of each treatment plan, a hypothalamic contour was retrospectively added in each case. For patients treated with Linac-based 3D-CRT, the treatment plan was restored into the Xio radiation planning system and the hypothalamic contour was added to the structure set, such that dose–volume histogram (DVH) data for the hypothalamus could be acquired. For patients treated with TomoTherapy, plans were restored into both Xio (to allow the hypothalamic contour to be added) and the TomoTherapy planning system (to recover the dose data itself) and fused into Focal (Elekta AB, Stockholm, Sweden) such that dose data from the original plan could be applied to the new hypothalamic contour. For patients treated with gamma knife SRS, the hypothalamic contour was added in Leksell Gamma Plan such that DVH data could be analyzed. Contouring within Gamma Plan was performed directly onto the pre-treatment MRI data set originally acquired using the MRI T1 sequence with contrast. Contouring was performed on pre-radiotherapy MRI T1 weighted images.

After restoration of all plans and construction of the hypothalamic contour was complete, the following parameters were determined: target volume, prescription dose, volume of the hypothalamus, volume of the hypothalamus receiving at least 12 Gy (V12Gy), Mean hypothalamic dose (DMean Hypo), Maximum hypothalamic dose (DMax Hypo), and the Biologically Equivalent Dose at 2 Gy/Fx for α/β = 3 (BED2Gy).

### Hypothalamic contouring

**Figure 1:**
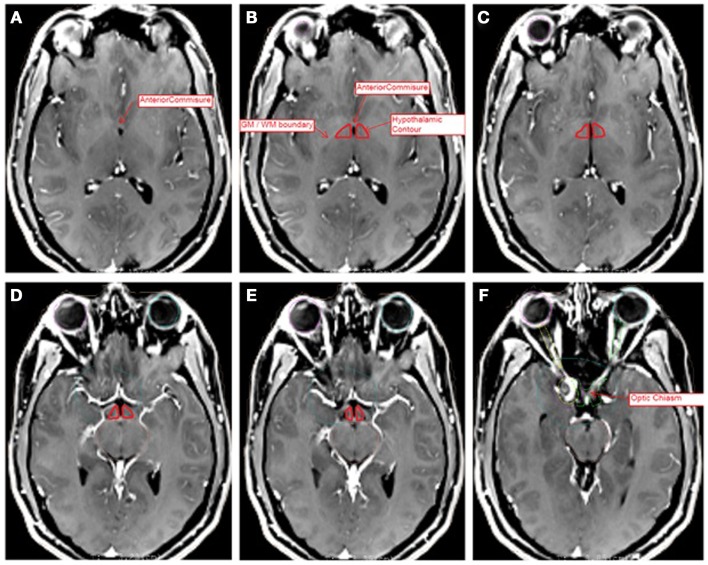
**Representative slices of MRI T1 + C images registered to CT data sets for hypothalamic volume contouring**. Slices A (superior most) through F (inferior most) depict several anatomic landmarks for the delineation of the hypothalamic contour (red). **(A)** Depicts the superior most slice, at the level of the anterior commissure. **(B–E)** Depict the hypothalamic contour, bounded by the white matter tracts laterally, the third ventricle medially, the CSF space of the suprasellar cistern anteriorly, and the level of the interpeduncular fossa posteriorly. **(F)** Depicts the level of the optic chiasm, at which the contour ends. The contour has been enhanced for ease of visibility.

The hypothalamic contour was created using MRI-based anatomic landmarks representing surrogate boundaries for the hypothalamus itself. Contouring was performed with the assistance of an MCW neuroradiologist, and contours were reviewed by the senior author (Joseph A. Bovi). Previously published MRI Atlases of the hypothalamus were referenced to identify anatomic landmarks as boundaries for the hypothalamic contour (10, 11). The contouring procedure was also in accordance with a previously published hypothalamic contouring method (8). The hypothalamic contour was a polygonal structure consisting of two separated volumes on each side of the third ventricle or CSF space. The superior most boundary of the contour was set at the axial slice containing the anterior commissure (AC). The inferior most boundary was set at the axial slice containing the OC. Anteriorly, the boundary consisted of the anterior aspect of the third ventricle or the visible edge of the CSF space within the suprasellar cistern. Posteriorly, the contour was drawn to the level of the interpeduncular fossa. The medial border consisted of the third ventricle or the visible CSF space. Laterally, the contour was bounded by the optic white matter tracts or the internal capsule. Representative slices are depicted in Figure 1.

### Endocrine evaluation

Baseline endocrine evaluation was performed on all analyzed patients under the direction of MCW neuroendocrinologists. Each patient had baseline pre-radiotherapy endocrine labs drawn, as well as post-radiotherapy follow-up labs at 6–12 month intervals after treatment indefinitely. Standard endocrine laboratory studies included evaluation of PRL, GH which was evaluated through IGF-1, thyroid studies (TSH and free T4), ACTH which was evaluated with eight AM fasting cortisol, ACTH, and eight AM fasting cosyntropin stimulation test, and gonadotropin function evaluated with estradiol or testosterone, LH and FSH or normal menstrual status in premenopausal women. Reference ranges were as follows: PRL (2.8–23.3 ng/mL female, 4.0–15.2 ng/mL male), IGF-1 (reference range varies with age), TSH (0.45–4.5 μIU/mL), free T4 (0.82–1.77 ng/dL), eight AM cortisol (>10 μg/dL), ACTH (7.2–63.3 pg/mL), FSH (male, 1.5–12.4 mIU/mL, female post menopause 25.8–134.8 mIU/mL, female pre-menopause with normal menstrual cycles considered normal, reference ranges are phase dependent), LH (male, 1.7–8.6 mIU/mL, female post menopause 7.7–58.5 mIU/mL, female pre-menopause with normal menstrual cycles considered normal, reference ranges are phase dependent). Laboratory reference ranges varied slightly during the study period; however, all laboratory values were referenced to the normal ranges listed at the time of lab draw. Pre-radiotherapy deficit was defined as any laboratory values below the reference ranges or any administration of hormone replacement therapy prior to the start of radiotherapy. Post-radiotherapy endocrinopathy in any axis (PRL, GH, ACTH, TSH, or FSH/LH) was defined as laboratory values on at least two separate lab draws below the reference range identified on a follow-up evaluation, or administration of hormone replacement therapy (such as levothyroxine, hydrocortisone/prednisone, human GH, estrogen/progesterone, or testosterone) as judged necessary by the treating neuroendocrinologist. In addition, PRL elevation post-radiotherapy in a previously non-PRL secreting adenoma was categorized as an endocrine abnormality. Any endocrine laboratory value outside the reference range on a single assessment that subsequently normalized without the administration of hormone therapy was not considered to constitute an endocrinopathy.

### Statistical analysis

With respect to endocrine evaluation, new HP-axis deficits that were not present prior to radiotherapy were considered the primary endpoint. HP-axis event curves using the Kaplan–Meier method were performed, with a new endocrinopathy in any of the five endocrine axes (GH, PRL, ACTH, TSH, or Gonadotropin) constituting an event in the analysis. If multiple endocrinopathies occurred within the same patient, the first endocrinopathy was used for the event curve analysis. Univariate analysis of event free survival by treatment modality was performed using the log-rank test. With respect to dosimetric evaluation, parameters analyzed included hypothalamic mean dose (DMean Hypo), hypothalamic maximum dose (DMax Hypo), and the percentage of hypothalamic volume receiving ≥12 Gy (V12Gy). Dosimetric comparisons by treatment modality for all three groups were performed using one-way ANOVA. TomoTherapy comparison with Linac-based therapy was performed using the Exact Wilcoxon Rank Sum test. Statistics were performed in consultation with the MCW department of Biostatistics using SAS statistics software version 9.3 (The SAS Institute, Cary, NC, USA) and significance was determined by a *p* < 0.05.

## Results

### Dosimetric comparisons

Dosimetric data are presented in Table 2. With respect to the fractionated techniques there was no difference between the Linac-treated group and the TomoTherapy group in terms of target volume (12.7 and 24.5 cc, respectively), prescription dose (50.4 and 50.68 Gy, respectively), volume of the hypothalamus (0.81 and 0.93 cc, respectively), and BED 2 Gy (48.4 and 49.1 Gy, respectively). When comparing the fractionated techniques, there was an improvement in the TomoTherapy group over the Linac group with respect to the hypothalamic V12Gy (76 vs. 100% *p* = 0.004), DMean (44.8 vs. 26.8 Gy *p* = 0.02), and DMax (49.8 vs. 39.1 *p* = 0.04). The gamma knife group was superior to both fractionated techniques in terms of DMax (1.6 Gy) and V12Gy (0%), however DMean for the gamma knife group was not calculated. The hypothalamic volume of the gamma knife group was smaller than that of either of the fractionated groups, likely reflecting differences in the contouring mechanism and the slice thickness of the MRI images available for the Gamma Plan system.

**Table 2:** **Dosimetric parameters by treatment modality**.

Parameter	Linac	Tomo Therapy	Gamma knife	*p*
Target volume (cc)	12.7	24.5	3.0	0.7
Hypothalamic volume (cc)	0.8	0.9	0.5	0.28
V12Gy Hypo	100%	76%	0%	0.004
DMean Hypo	44.8 Gy	26.8 Gy	NA	0.02
DMax Hypo	49.8 Gy	39.1 Gy	1.6 Gy	0.04
BED2 α/β 3	48.4 Gy	49.1 Gy	60.6 Gy	0.64
Mean prescription dose	50.4 Gy	50.7 Gy	15.8 Gy	0.66

*BED2 α/β 3, biologically equivalent dose at 2 Gy per fraction for an α/β ratio of 3*.

All gamma knife parameters were significantly different than the respective fractionated radiotherapy parameters by the exact Kruskal–Wallis Test. *p* Values reflect comparison of Linac vs. Tomotherapy by the exact Wilcoxon Rank Sum Test.

### Patient survival, local control, and endocrine outcomes

All patients were alive and disease free during the study period after completion of fractionated EBRT or gamma knife radiosurgery. No patient exhibited local recurrence as assessed by MRI imaging during follow-up.

Prior to radiotherapy, 28 of 33 (84.8%) of patients had at least one baseline endocrine deficit. Post-radiotherapy for all groups, new endocrine deficits occurred in 13 of 27 (48%) of patients eligible for analysis. A total of 20 new endocrine deficits were identified, with some patients developing more than one endocrinopathy. Deficits by treatment modality were as follows: in the Linac-based 3D-CRT treated group 8 of 11 (72%) patients developed at least one new endocrinopathy, whereas this occurred in 4 of 8 (50%) patients treated with TomoTherapy IMRT and 1 of 8 (12.5%) patients treated with gamma knife SRS. Specific endocrine deficits by treatment modality are displayed in Table 3. Of 20 new endocrine deficits identified, 5 (25%) each involved GH, TSH, and PRL, 2 (10%) involved ACTH, and 3 (15%) involved LSH/FSH.

**Table 3:** **Specific endocrine deficits by treatment modality**.

Hormone abnormality	Linac	Tomo Therapy	Gamma knife	Total (%)
GH	4	1	0	5 (25)
PRL (↓)	1	0	0	1 (5)
PRL (↑)	2	1	1	4 (20)
ACTH	2	0	0	2 (10)
TSH	3	2	0	5 (25)
LSH/FSH	3	0	0	3 (15)
Total	15	4	1	20 (100)

**Figure 2:**
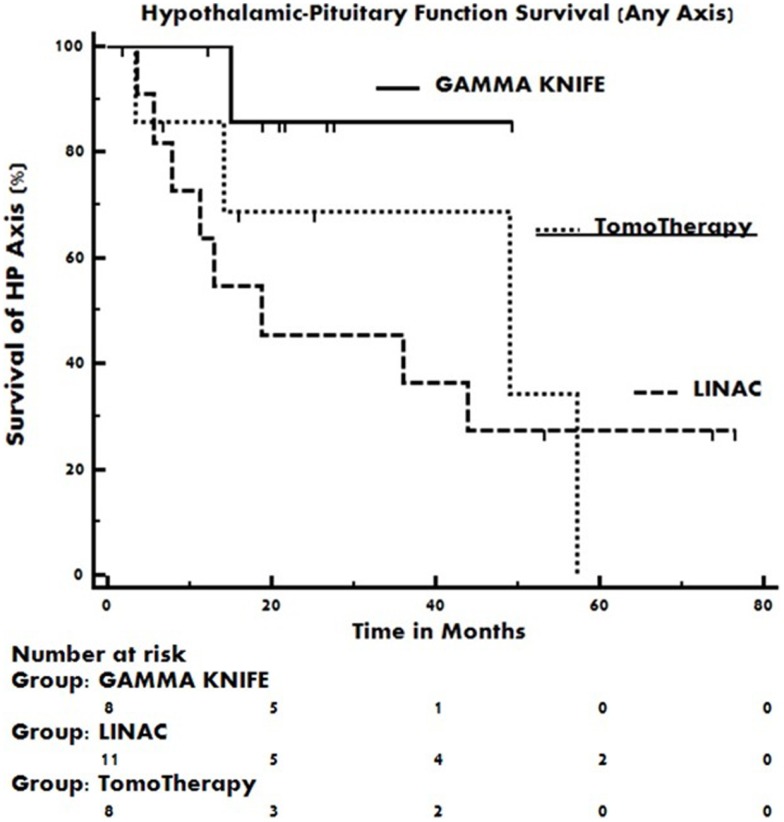
**Kaplan–Meier event curves of pre-radiotherapy intact hypothalamic–pituitary axes as a function of time post-radiotherapy**. Occurrence of any new endocrinopathy constitutes an event in the plot. Event curve hazard ratios do not differ statistically by the log-rank test.

The median event free duration of the functioning pre-radiotherapy hypothalamic–pituitary axes were 18.7 months in the Linac 3D-CRT treatment group, 48.9 months in the TomoTherapy IMRT treatment group, and was not reached in the gamma knife SRS treatment group. The pre-radiotherapy intact endocrine axis event curves for each group are displayed in Figure 2. Event curve separation was not statistically significant by the log-rank test (*p* = 0.36).

## Discussion

One of the most common complications of treatment for pituitary adenomas is endocrine dysfunction, and for this reason long term follow-up is critical to optimize patient outcomes. This risk as it relates to radiotherapy is a phenomenon that depends on dose to the HP-axis, the volume of irradiated tissue, the time interval after irradiation, and underlying HP-axis pathology (12). Patients undergoing both fractionated EBRT and SRS are subject to this risk. Typically endocrinopathies manifest within the first 3 years of treatment, however they can also occur much later (13). Despite the consistent finding that irradiation of pituitary tumors places the patient at risk for hypopituitarism, reports of the actual incidence of treatment-associated endocrine deficits vary widely among both fractionated and radiosurgical series (14, 15). The reason for this heterogeneity is likely multifactorial and involves numerous issues such as the type and extent of surgery prior to radiotherapy, the pre-treatment effect of the tumor itself on the HP-axis, the type of radiotherapy technique, the follow-up interval, and the definition of endocrine dysfunction used. Various definitions of post-treatment endocrine dysfunction have been employed such as the use of strict laboratory cutoff values vs. the administration of hormone replacement therapy, or a combination thereof (16–19).

In this study, the definition of new endocrine deficit included both a consistently abnormal laboratory value as well as the administration of hormone replacement therapy in order to capture these two issues. By doing so, however this study may reflect relatively higher rates of endocrinopathy in comparison to other series. Additionally, it is uncommon to report post-radiotherapy PRL elevation as a new HP-axis endocrinopathy, as this is unlikely to cause symptoms or require intervention. However, this particular abnormality does give insight into the mechanism of dysfunction, as the hypothalamus is known to exert a tonic inhibitory effect on the secretion of PRL by the anterior pituitary through the neurotransmitter dopamine (20, 21). Presumably, a post-radiotherapy rise in PRL would represent damage sustained by the hypothalamus reflected by the inability to maintain this tonic inhibition, and in certain circumstances can be opposed through the use of a dopamine agonist (22).

The exact contribution of the hypothalamus to post-radiotherapy anterior pituitary endocrine deficits is not entirely elucidated, however it is postulated that endocrine changes resulting from hypothalamic damage may occur within a shorter time frame than those associated with damage to the pituitary itself. Agha et al. analyzed HP-axis deficiencies in adults treated with radiotherapy for non-pituitary brain tumors and found that mild to moderate hyperprolactinemia occurred at a shorter median time (33 months) in comparison to GH, ACTH, or gonadotropin deficiency (100 months), possibly reflecting an earlier manifestation of hypothalamic dysfunction in comparison to pituitary dysfunction (18).

Studies of GH deficiency in pediatric patients treated for primary brain tumors as well as total body irradiation (TBI) have revealed that the hypothalamus plays an important role in the pathogenesis of this disorder. Merchant et al. investigated the relationship of the dose to the hypothalamus in the development of GH deficiency and determined that a dose of 16.1 Gy to the hypothalamus would constitute the mean dose required to induce a 50% risk of GH deficiency at 5 years post-radiotherapy (23). GH is known to be the most susceptible endocrine axis to radiotherapy-induced dysfunction, and hypothalamic doses as low as 12 Gy may result in impaired GH release (8). Although susceptibility of the hypothalamus to radiation-induced dysfunction may be more pronounced in children, the same phenomenon has been noted in adult patients as well. Feigl et al. investigated the hypothalamic dose received by 108 patients treated with gamma knife SRS for pituitary adenomas and found that the mean dose was higher in patients with new endocrinopathies than in those without (1.3 vs. 0.8 Gy) (17).

Overall endocrine dysfunction likely reflects the combined effect of damage to both structures (5, 7, 14). In clinical practice, however it is not useful or cost effective to routinely differentiate secondary endocrine deficiencies from tertiary endocrine deficiencies (i.e., anterior pituitary vs. hypothalamic) as this distinction would not typically alter management.

Given the role played by the hypothalamus in the development of radiation-induced hypopituitarism, dose monitoring to this structure with the aim of maximal avoidance is a rational objective. In this study, the parameters DMax Hypo and DMean Hypo were chosen as dose monitoring parameters analogous to those commonly employed for other treatment planning organs at risk (OAR). The parameter V12 Hypo (the volume of the hypothalamus receiving 12 Gy) was chosen as possibly reflective of a “threshold dose” in the fractionated setting, as it has previously been shown through TBI data that dysfunction of the most sensitive HP-axis, GH, is rare with exposure of <12 Gy (9). In addition, in the radiosurgical setting, the volume of brain receiving >12 Gy has been described in QUANTEC as a marker for increased normal tissue complication risk (24).

In this study, dose sparing to the hypothalamus was considerably more favorable in the gamma knife SRS group over the fractionated techniques. This was an expected result, given the highly conformal nature of the gamma knife system and steep dose gradients associated with this technique. It is important to note that to be eligible for this procedure patients must meet certain requirements in terms of tumor size and distance from the optic structures.

With respect to the fractionated techniques, helical TomoTherapy-based IMRT exhibited improved hypothalamic dose sparing in comparison to Linac-based 3D-CRT, likely in part due to the characteristic anterior vertex field of the 3D plans, which projects through the hypothalamus. Improved conformality through IMRT inverse planning is not unique to TomoTherapy, as Linac-based IMRT techniques are capable of producing improved dosimetric profiles in comparison to the Linac-based 3D technique; however this issue was not addressed during the study period as most treating physicians were more comfortable with the use of TomoTherapy IMRT in this setting. The endocrine outcomes of the patients in each of these three groups trended toward improved HP-axis preservation paralleling the hypothalamic sparing properties of the techniques used, however larger patient sample and longer follow-up would likely be required to further clarify the association. Furthermore, routinely incorporating the hypothalamic contour may allow for improved conformal avoidance with either fractionated 3D-CRT or IMRT techniques.

## Conclusion

In patients treated post-operatively for pituitary adenomas, hypothalamic dose sparing was best achieved using gamma knife-based radiosurgery. With respect to fractionated radiotherapy, TomoTherapy IMRT provides improved hypothalamic dose sparing over Linac-based 3D-CRT. Efforts to minimize hypothalamic dose by monitoring parameters such as DMax Hypo, DMean Hypo, and V12Gy may improve endocrine outcomes post-radiotherapy.

## Conflict of Interest Statement

The authors declare that the research was conducted in the absence of any commercial or financial relationships that could be construed as a potential conflict of interest.
